# Single-base resolution methylomes of upland cotton (*Gossypium hirsutum* L.) reveal epigenome modifications in response to drought stress

**DOI:** 10.1186/s12864-017-3681-y

**Published:** 2017-04-13

**Authors:** Xuke Lu, Xiaoge Wang, Xiugui Chen, Na Shu, Junjuan Wang, Delong Wang, Shuai Wang, Weili Fan, Lixue Guo, Xiaoning Guo, Wuwei Ye

**Affiliations:** 1 State Key Laboratory of Cotton Biology/Institute of Cotton Research of Chinese Academy of Agricultural Sciences/Key Laboratory for Cotton Genetic Improvement, Anyang, 455000 Henan China; 2 College of Agronomy, Xinjiang Agricultural University, Urumqi, 830052 China

**Keywords:** Methylomes, Upland cotton, Epigenome modifications, Long non-coding RNAs, Drought stress

## Abstract

**Background:**

DNA methylation, with a cryptic role in genome stability, gene transcription and expression, is involved in the drought response process in plants, but the complex regulatory mechanism is still largely unknown.

**Results:**

Here, we performed whole-genome bisulfite sequencing (WGBS) and identified long non-coding RNAs on cotton leaves under drought stress and re-watering treatments. We obtained 31,223 and 30,997 differentially methylated regions (representing 2.48% of the genome) after drought stress and re-watering treatments, respectively. Our data also showed that three sequence contexts, including ^m^CpG, ^m^CHG, ^m^CHH, all presented a hyper-methylation pattern under drought stress and were nearly restored to normal levels after the re-watering treatment. Among all the methylation variations, asymmetric CHH methylation was the most consistent with external environments, suggesting that methylation/demethylation in a CHH context may constitute a novel epigenetic modification in response to drought stress. Combined with the targets of long non-coding RNAs, we found that long non-coding RNAs may mediate variations in methylation patterns by splicing into microRNAs. Furthermore, the many hormone-related genes with methylation variations suggested that plant hormones might be a potential mechanism in the drought response.

**Conclusions:**

Future crop-improvement strategies may benefit by taking into account not only the DNA genetic variations in cotton varieties but also the epigenetic modifications of the genome.

**Electronic supplementary material:**

The online version of this article (doi:10.1186/s12864-017-3681-y) contains supplementary material, which is available to authorized users.

## Background

An increasing number of documents have evidenced the importance of DNA methylation in the process of plant growth and development, plant defense, and the response to adversity stress, including salt, drought and heavy metal stress. Naturally occurring modifications in a single gene locus in plants may yield heritable morphological changes without alteration of the DNA sequence [1, 2], and some of these modifications may even be passed down through several generations. *Arabidopsis*, a model plant, is the first plant for which a whole-genome methylome map was deciphered, for its compact genome and rapid cycle [3–5]. Cotton has always been known as a primary model plant for studying genome polyploidization [6], cell fate determination, cell elongation and cell wall formation [7]. Currently, the genomes of diploid cottons *G. arboreum* (AA) and *G. raimondii* (DD) and complex allotetraploid *G. hirsutum* L. (AADD) have all been completed [8–10], and high-quality reference genomes and improving sequencing technologies may better ensure the whole-genome methylome analyses of cotton and some other moderate-sized crop genomes, such as the recent discovery of the tomato fruit methylome and of hypomethylation in the rice endosperm [11, 12]. Here, we investigate whether whole-genome epigenome reprogramming occurs during the biologically important processes of cotton in response to drought stress.

The response to drought stress in plants is a complicated process, involving several genes and metabolic networks, and DNA methylation is one of them. Publications have shown that drought can induce alterations of the DNA methylation locus and patterns in plants with variety specificity, tissue specificity and stress specificity [13–15]. The establishment and maintenance of DNA methylation patterns that result in gene expression modulation is one of the key steps in epigenetic regulation during normal growth and developmental programs. Documents about transcriptome analysis and drought-response miRNA identification and functional analysis have been publicated [16–18]. Recent discoveries have identified long non-coding RNAs (lncRNAs, defined as transcripts of >200 bp without protein-coding capacity) as new important players in DNA methylation regulation [19]. A recent study published in *Nature* showed a novel lncRNA arising from the *CEBPA* gene locus (termed *ecCEBPA*) that is critical for regulating DNA methylation at this site through the binding of *ecCEBPA* with DNA methyltransferase1, DNMT1 [20]. Interestingly, a robust increase was observed in the levels of DNA methylation of the *CEBPA* promoter region following the depletion of this non-coding transcript [20]. However, the alterations of the DNA methylome and the possible roles of lncRNAs in regulating the occurrence of methylation are still unclear under drought conditions in cotton.

Here, we provide a high-resolution and high-coverage map comprising the methylation status of individual cytosines throughout the cotton genome in response to drought stress. On the basis of the possible regulatory functions of lncRNAs, and to investigate the roles of lncRNAs in adjusting the epigenome modifications, we integrated the DNA methylome and lncRNAs. Based on this resource, future studies can use low-coverage methylome sequencing to determine the impact of differentially methylated regions on gene expression, chromosome biology, and transgenerational inheritance. For example, this will allow breeders to determine the contribution of epigenetic modifications to phenotypic variations [21], and the breeders can utilize a form of “epigenetic selection” to analogously select the individuals with desired epigenomic patterns in breeding programs.

## Results

### Genome-wide patterns of DNA methylation

**Fig. 1:**
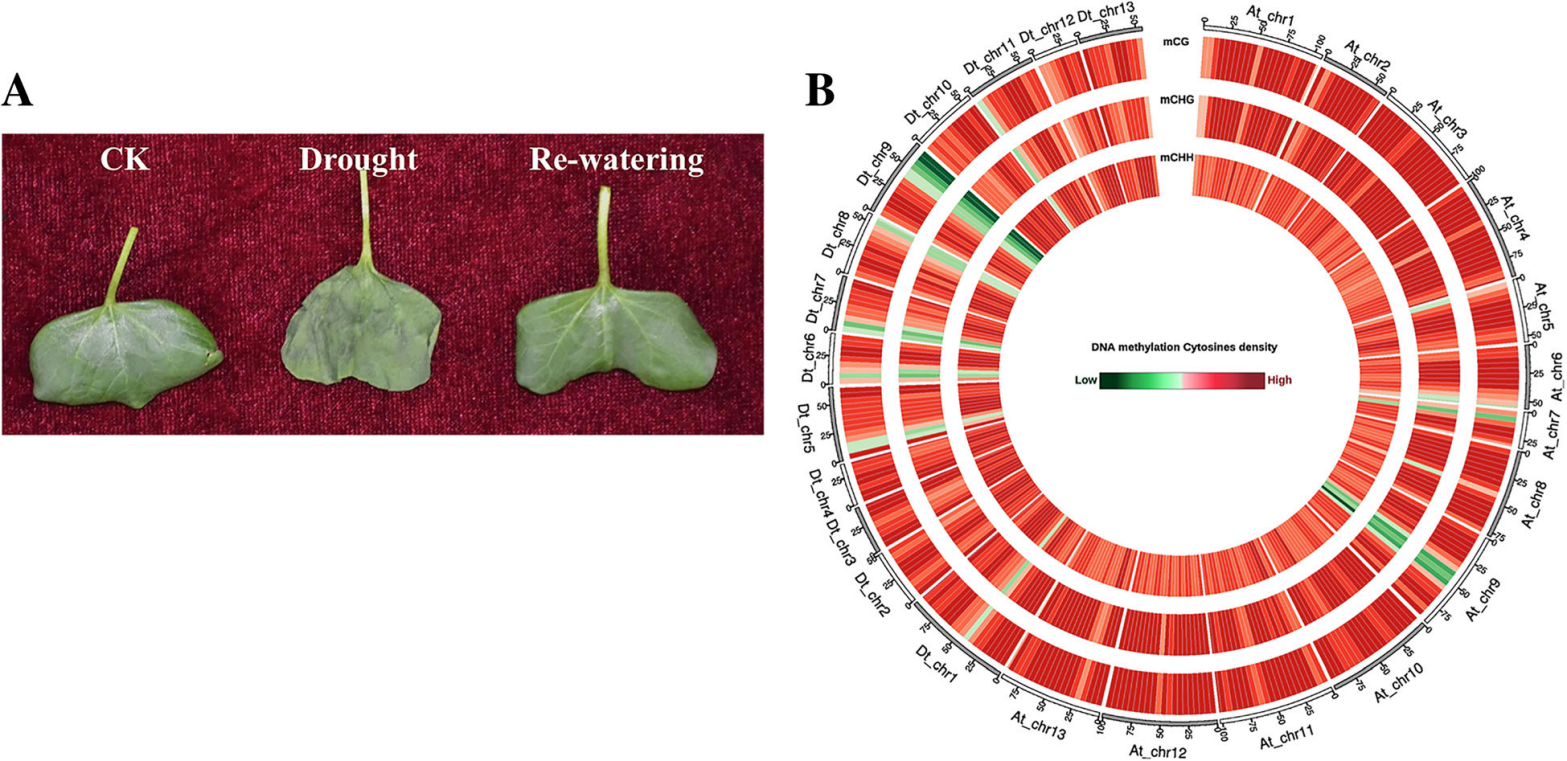
Epigenome of *Gossypium hirsutum* L. **a** Morphological changes of cotyledons under different treatments. **b** Density plot of 5-methylcytosine in sequence contexts (mCG, mCHG and mCHH where mC signifies 5-methylcytosine, and H represents A, C or T). The circles from the outside to inside represent mCG, mCHG, mCHH, respectively

Bisulfite treatment of DNA, termed the “gold standard” of DNA methylation research, can convert unmethylated cytosines into uracils while leaving methylated cytosines unchanged, which allows the generation of “methylomes” at single-base resolution [22]. The bisulfite-converted DNA of drought-tolerant cotton variety ZhongH177 under different treatments, including CK, drought (relative water content (RWC) reached approximately 7% in pots) (Fig. 1a), and re-watering, was sequenced with Illumina Hi-Seq instruments at a coverage that provided information on the methylation states of individual cytosines with high confidence. The percentage of methylated cytosines varied depending on the local sequence context (C, CG, CHG and CHH) and the external treatments (CK, drought and re-watering). The results of WGBS are listed in Table 1. We obtained 228,002,142 and 256,948,717 and 253,873,809 reads after CK, drought and re-watering stresses, respectively. In addition, the mapping rate was 65.28, 67.68 and 67.51%, respectively, showing that the method and the results have high reliability and accuracy. The distribution of methylation sites in each chromosome was analyzed (Fig. 1b and Additional file 1: Figure S1, Additional file 2: Figure S2 and Additional file 3: Figure S3). Among the methylated cytosines, we found that more than half of them were located in asymmetric CHH contexts (Table 1). The total ^m^C in the whole genome under three environments (CK, D and re-watering) was 27.99, 32.34 and 29.95%, respectively, showing an up-regulated methylation level after drought stress and a re-down-regulated trend after recovery from drought stress by re-watering compared with CK. The duplication rate (the percentage of repetitive sequences in all clean sequencing reads) increased approximately 2% after drought stress and re-watering (Table 2), which showed that repetitive sequences may be important in response to drought stress through the alterations of the methylation level and state. Interestingly, we found that methylated cytosines in ^m^CpG, ^m^CHG, ^m^CHH contexts all showed a hyper-methylation pattern after drought stress compared with CK and re-watering, but the alterations in the CHH contexts were more significant than the alterations in other contexts. This finding suggested that methylation levels in asymmetric CHH contexts were dynamically changing with external environments and were mostly correlated with environments. In symmetric CG and CHG contexts, more than half of these cytosines were methylated while only a small proportion were methylated in the CHH context (Table 2).

**Table 1:** Bisulfite sequencing summary

Samples	Total reads	Mapped reads	Mapping rate (%)	^m^C percent (%)	^m^CG percent (%)	^m^CHG percent (%)	^m^CHH percent (%)
CK	349,288,166	228,002,142	65.28	201,543,688 (100)	47,879,971 (23.76)	45,874,296 (22.76)	107,789,421 (53.48)
D	379,630,307	256,948,717	67.68	232,876,499 (100)	50,588,873 (21.72)	48,445,561 (20.80)	13,3842,065 (57.47)
Re_W	376,067,028	253,873,809	67.51	215,698,912 (100)	49,367,000 (22.89)	47,336,957 (21.95)	118,994,955 (55.17)

Total reads are the clean sequencing reads after the data filtration. Mapping rate are the reads with unique positions on reference genome after alignments. ^m^C, ^m^CG, ^m^CHG, ^m^CHH percent (%) are the percentages of methylated ^m^C in each context in all methylated cytosines from the whole genome

**Table 2:** Statistical results of methylated cytosines in different contexts

Samples	Duplication rate (%)	^m^C percent (%)	^m^CpG percent (%)	^m^CHG percent (%)	^m^CHH percent (%)
CK	10.70	27.99%	58.42%	53.77%	19.50%
D	12.48	32.34%	61.71%	56.78%	24.21%
Re_W	12.24	29.95%	60.22%	55.48%	21.52%

Duplication rate is the percentage of repetitive sequences in all clean sequencing reads. ^m^C percent (%) is the percentage of methylated cytosines in all cytosines from the whole genome. ^m^CG, ^m^CHG, ^m^CHH percent (%) are the percentages of methylated ^m^CG, ^m^CHG, ^m^CHH in all corresponding cytosines context (C, CG, CHG, CHH and H represents A, C, T) in genome, respectively

### Cytosine DNA methylation may have sequence preference

**Fig. 2:**
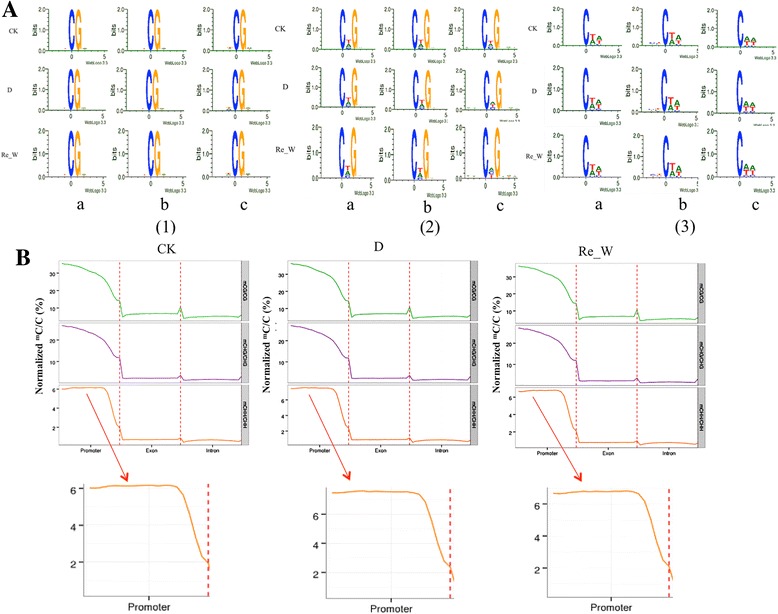
Sequence preference of Cytosine DNA methylation and differentially methylated cytosines in gene elements. (A) Weblogo analysis of bases around ^5m-^C in different sequence contexts. X axis represents bases position and Y axis represents the degree of base enrichment. The position of C was defined as 0. (1), (2), (3) represent CG, CHG and CHH context, respectively. a, b, c represent all ^m^C sites, high methylation sites and low methylation sites, respectively. In CG context, we defined that methylation level of high methylation level sites was >75% and others were low methylation level sites. In non-CG context, we defined that methylation level of high methylation level sites was >25% and others were low methylation level sites. (B) Methylation level on different genome elements. X axis represents different gene elements, and Y axis represents methylation levels. Each element of each gene was divided into 20 bins and the average of all bins in each Gene element was obtained. Different colours represent different sequence contexts

Studies showed that the occurrence of cytosine methylation was associated with its nearest sequence context [4]. Therefore, we used a tool named Logo Plots, which explores the sequence information of a methylated site or its nearby regions, to help us to understand the preference of methylated sites. The method is described in the “Methods” section. Among all methylated ^m^C sites, the sequence preference around ^m^C sites varied with the CG context and non-CG context (Fig. 2a). In the symmetric CG context, ^m^C frequently occurred at TCGA sequences, regardless of the presence of low-methylation-level sites or high-methylation-level sites. In the CHG context, we found that the ^m^C of high methylation levels occurred in CTG sequences, whereas at low methylation levels, ^m^C appeared in CTG or CAG sequences, which may be caused by drought stress and re-watering treatments. However, in the CHH context, low-methylation-level ^m^C sites often occurred in the CAA sequence context, and high-methylation-level ^m^C sites occurred in the CTA context. Therefore, we inferred that most high-methylation-level ^m^C sites regularly occurred in the CTN (N represents A, T, C, G) context, while low-methylation-level ^m^C sites occurred in the CAN (N represents A, T, C, G) context. Studies in humans suggest that CHG cytosine in the TACAG context is more easily methylated, and this type of methylation is always found at splicing sites [23]. Along with the alterations of methylation levels, the frequency of CG dinucleotide and its nearest sequences is also changed. However, this is not always consistent in all creatures. In humans, the methylation extent of the CpG dinucleotide is not closely related to its nearest sequence context; however, in terms of CHH and CHG, T and A are always upstream and downstream of cytosines with a higher methylation level [24].

### Differentially methylated regions (DMRs) responding to drought stress

Based on the annotation of gene elements (including promoters, exons and introns) [25], we compared the methylation levels in each context in each gene element (Fig. 2b), which can help to identify the functions of methylation alterations in each element in response to drought stress. We found that promoters displayed significantly higher levels of methylation than did exons and introns, suggesting that the methylation levels of C may be correlated with gene elements. In each gene element, the frequencies of each context, including CG methylation, CHG methylation and CHH methylation, were disparate, and CG methylation accounted for approximately half of the total ^m^C, which suggested that CG methylation was the most important methylation pattern in the three gene elements. Previous publications have showed that methylation most frequently occurs in the so-called CpG islands in the 5’ regulatory gene regions (promoters) [26]. We then compared the methylation levels of different contexts in promoter, exon and intron pre- and post-drought stress, and minuscule changes were found, except for CHH methylation (Fig. 2b). Interestingly, we found that the CHH methylation in the promoter region exhibited an up-regulated pattern under drought stress compared with the control and a slightly down-regulated pattern after the re-watering treatment compared with drought stress. This also suggested that the CHH methylation level is dynamic with environments and that CHH methylation may be very closely correlated with drought stress. Studies have shown that methylation in the CHH context increases compared with the CG and CHG context, and this CHH methylation can be explained by genes promoting de novo methylation on flanking intergenic chromatin (particularly within a few kb of gene starts, that is, always in promoter regions) [27]. Consequently, we inferred that the mechanism through which methylation is altered in promoter regions can be correlated with genes promoting de novo methylation to regulate gene expression in response to drought stress.

**Fig. 3:**
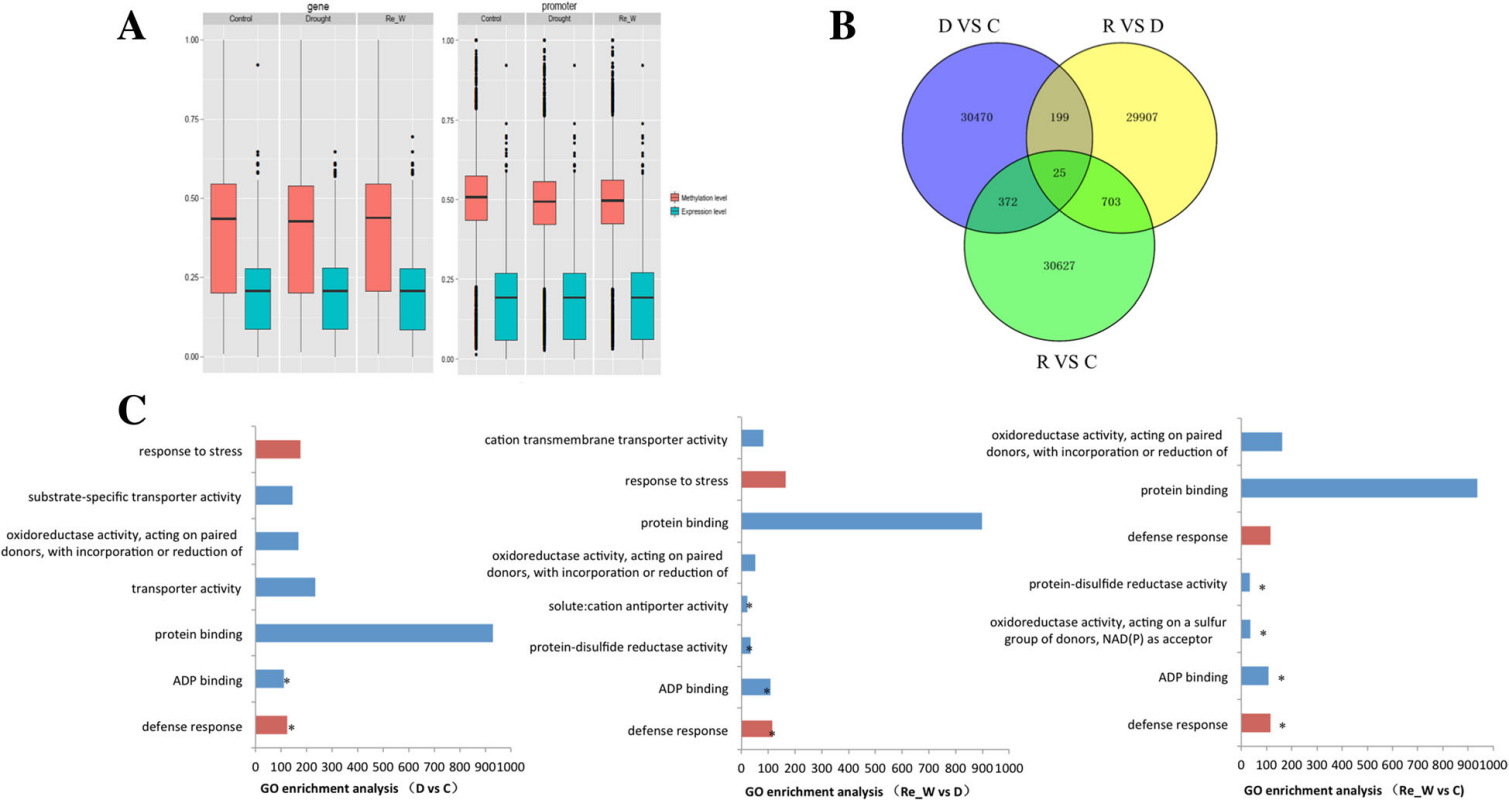
Differentially methylated regions and GO analysis. **a** Methylation and expression level on different genome elements. X axis represents different treatments (CK, drought stress and re-watering), and Y axis represents methylation levels or FPKM-log_ratio of genes. *Red* was methylation level and *green* was gene expression level. The calculation method used in the process was FPKM_log_ratio = log_10_(FPKM(k))/log_10_(max(FPKM)). **b** Number of DMRs under drought stress. **c** GO enrichment analysis of DMRs. GO analysis contains three branches: Cellular component, molecular function and biological process. *Red* represents biological process and *blue* represents molecular function. *Represents statistical significance level at P = 0.05

Increasing evidence has shown that the methylation and demethylation processes are always correlated with altered gene expression [28]. Therefore, we analyzed the influences of methylation changes on gene body and promoter regions, and the results are shown in (Fig. 3a). To analyze the relationship between DNA methylation and gene expression, the FPKM-log_ratio was used to uniformize the FPKM value of genes and to avoid a large range of gene expression levels. The results indicated that the range of methylation changes in the gene body region was large, while the promoter was small. Furthermore, the overall methylation level in the promoter was higher than that in the gene body region, which may be closely related to the gene functions. Combined with methylation variations and gene expression changes in the gene body and promoter regions, we found that gene expression could be influenced by DNA methylation changes. In the gene body region, the range of gene expression alterations was narrow compared with those of promoters, and there were some additional genes with a great difference in expression in the promoter region, which indicated that methylation in the gene body may influence gene expression levels to a relatively low and intensive level, while a higher methylation level in a promoter may yield a scattered scope of gene expression. Taken together, our results showed that methylation in the gene body and promoter region can suppress gene expression to some extent.

To assess the role of methylation in response to drought stress, we examined differentially methylated regions (DMRs) under drought stress compared with controls, which were always considered to participate in the process of gene regulation. We discovered 31,223 DMRs under drought stress compared with the control, and the number of DMRs decreased to 30,997 after the re-watering treatment (Fig. 3b), which indicated that some DMRs were dynamically changed along with the external environments and that, when the adversity was removed, the methylation sites would return to the normal pattern. We also performed a DMR length analysis in each chromosome (Additional file 4: Figure S4, Additional file 5: Figure S5 and Additional file 6: Figure S6) and found that the length of DMRs varied among 26 chromosomes, which may be correlated with the gene numbers, transposon numbers and certain other elements in each chromosome. To determine the role of DMRs during the response to drought stress, we performed Gene Ontology term enrichment analysis of differentially methylated genes (Fig. 3c). Genes involved in protein binding, ADP binding, defense response and transporter activity were highly enriched. Moreover, a GO analysis of hyper- and hypo- methylated genes showed that these genes with great changes in methylation levels or methylation patterns may be closely correlated with drought (Additional file 7: Figure S7 and Additional file 8: Figure S8), in turn suggesting that methylation alterations may be a mechanism to regulate gene expression in response to drought stress. The KEGG pathway analysis results showed that pathways such as ribosomes, RNA degradation, protein processing in the endoplasmic reticulum, and plant hormone signal transduction may be closely correlated with the response to drought (Additional file 9: Figure S9). Above all, we inferred that plants can change the methylation levels or patterns of certain genes in some pathways related to stress to regulate gene expression, which is a mechanism to respond to stress.

### Long non-coding RNAs (lncRNAs) may mediate the occurrence of DNA methylation

**Fig. 4:**
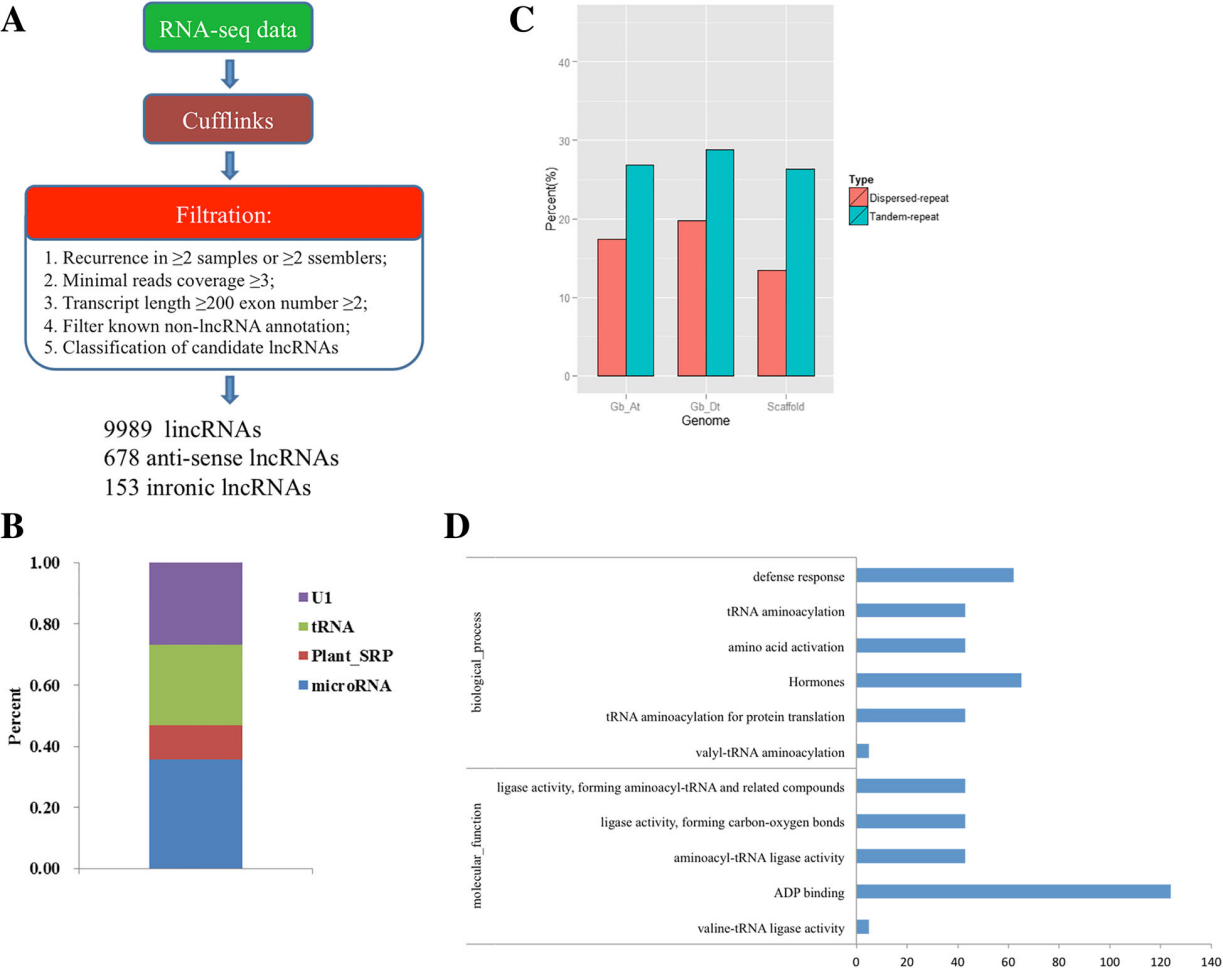
LncRNA results and the prediction of lncRNA-DMR related genes. **a** The filtration steps and products of lncRNAs. **b** Precursors prediction of lncRNAs. **c** Repetitive sequences and transposons analysis of lncRNAs. **d** GO analysis of lncRNA-DMR related genes by prediction

DNA methylation was the first discovered epigenetic mechanism [29, 30] and was reported to be regulated by lncRNAs [31]. To assess the role of lncRNAs in regulating DNA methylation changes in response to drought stress, we performed whole-genome screening and identification of lncRNAs responding to drought, and we obtained 9,989 lincRNAs, 678 anti-sense lncRNAs and 153 intronic lncRNAs through five strict filtration steps (Fig. 4a). The regulatory potential of lncRNAs has never ceased to amaze: from RNA catalysis to RNA-mediated splicing, RNA-based silencing, and RNA-directed DNA methylation [32]. LncRNAs are always the precursors of microRNAs and play a role by breaking into one or more microRNAs. Therefore, we conducted microRNA prediction of these identified lncRNAs with a threshold (E-value1 ≤ 0.05) (Fig. 4b), and the results indicated that approximately 40% of lncRNAs were the precursors of microRNAs.

In plants, DNA methylation typically occurs by RNA-directed DNA methylation (RdDM), which directs transcriptional gene silencing of transposons and endogenous transgenes, and RdDM is driven by non-coding RNAs (ncRNAs) [33]. Publications have reported that de novo DNA methylation can be directed by many long non-coding RNAs (80-nucleotide) and small interfering RNAs (24-nucleotide) [34, 35]. We also performed repetitive sequence and transposon analyses of lncRNAs (Fig. 4c), as many 20–24 nucleotide siRNAs were derived from repetitive sequences and transposons. Consequently, we speculated that it may be a regulatory mechanism through which long non-coding RNAs mediate the occurrence by splicing into microRNAs during the drought response process. Based on lncRNA-seq data, we screened the DMRs with a corrected *p*-value ≥ 0.05, and 514 target genes were identified with high confidence. The GO analysis of these genes is presented in Fig. 4d.

### DNA methylation may participate in the regulation of plant hormones

**Fig. 5:**
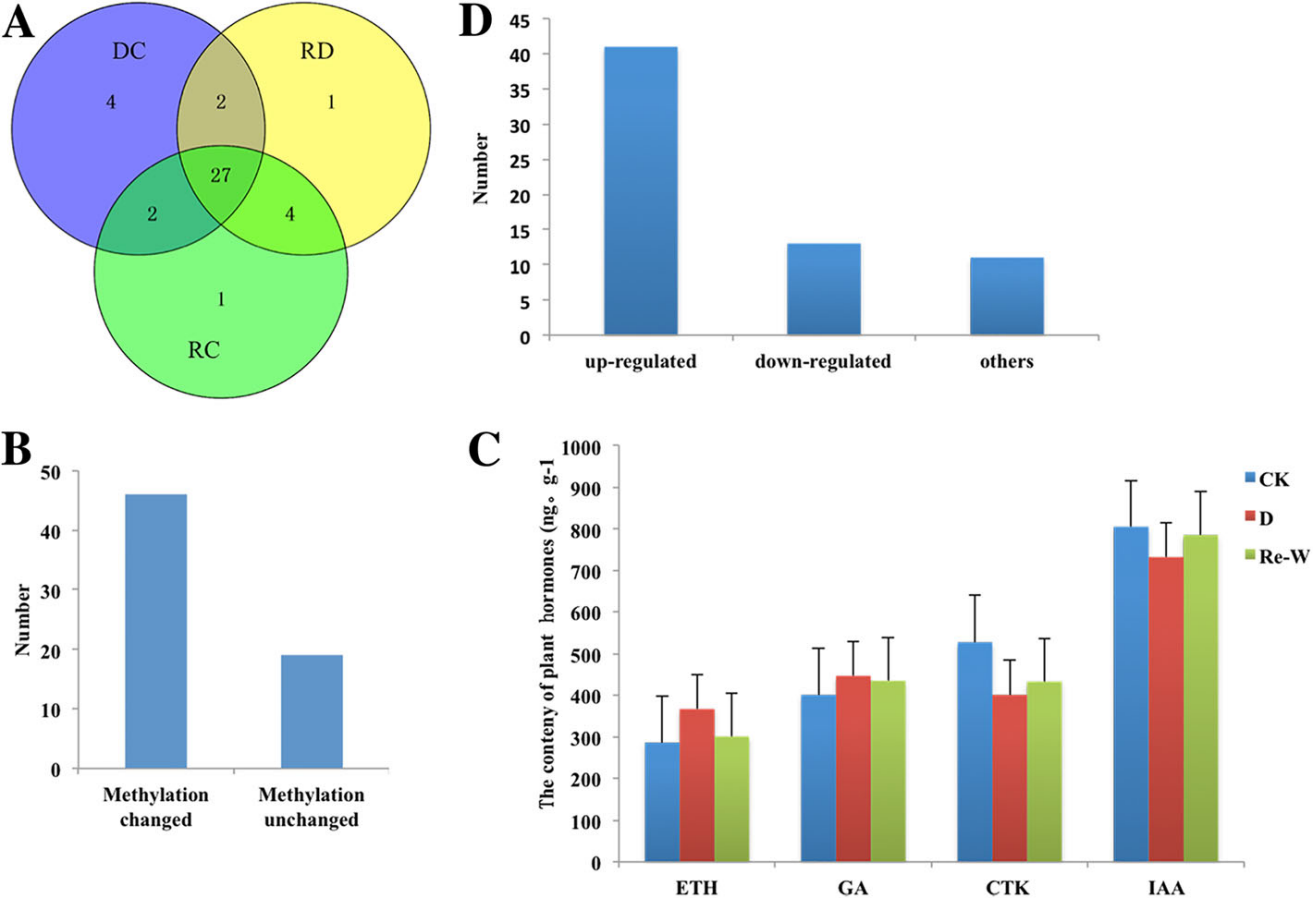
Regulation of plant hormones may be associated with DNA methylation. **a** Venn diagram of differentially expressed genes associated with hormones. DC, RD and RC represent differentially expressed hormone genes between drought and control, re-watering and drought, and re-watering and control. **b** Number of genes with methylation-state changed. **c** The content variations of plant hormones under drought stress and re-watering treatment. **d** Expression analysis of plant hormone-related genes with qRT-PCR

Based on the analysis of GO and KEGG pathways, we discovered 65 differentially methylated genes associated with hormones (Fig. 5a), of which, 6, 34, 9 and 16 were related to ethylene, auxin, gibberellin and cytokinin, respectively. To further investigate whether DNA methylation is involved in the regulation of hormone-related genes, we used methylation-specific PCR (MSP) to test the methylation changes pre- and post-drought treatment. Nearly 70.76% (46/65) of genes were induced to change their methylation state by drought stress (Fig. 5b), of which we found that approximately 66.67% (4/6), 55.56% (5/9), 43.75% (7/16) and 41.17% (14/34) of genes related to ethylene, gibberellin, cytokinin and auxin were induced to be de-methylated, which was consistent with the result of the content variations of plant hormones (Fig. 5c). To further examine the regulatory roles of DNA methylation on the hormone-related genes, we conducted the expression analysis of 65 genes with qRT-PCR, and the results showed that 83.07% (54/65) of these genes exhibited a marked change in expression, including 41 genes that were up-regulated and 13 genes that were down-regulated (Fig. 5d), which were almost in agreement with the methylation changes in the process. Zhong et al. showed that differentially methylated cytosines (DMCs) were mainly concentrated in regions 5′ upstream of genes and were therefore more likely to be associated with promoter regulatory regions [11]. To determine whether the epigenetic variations were associated with promoter regions, we screened the locations of these differentially methylated cytosines (DMCs) through BLAST analysis between control and drought-treated samples. We observed the enticement of DMCs (50/65) in promoter regions, which were similar to the methylation variations reported in *Arabidopsis thaliana* leaves [5, 36]. Consequently, this finding might indicate that DNA methylation in the promoter regions of hormone-related genes is most likely to be involved in the regulation of plant hormones (including ethylene, auxin, gibberellin and cytokinin), which is a possible mechanism for controlling plant hormones in the drought response and may contribute to the drought resistance of cotton.

### Methyltransferase inhibition could enhance drought resistance

**Fig. 6:**
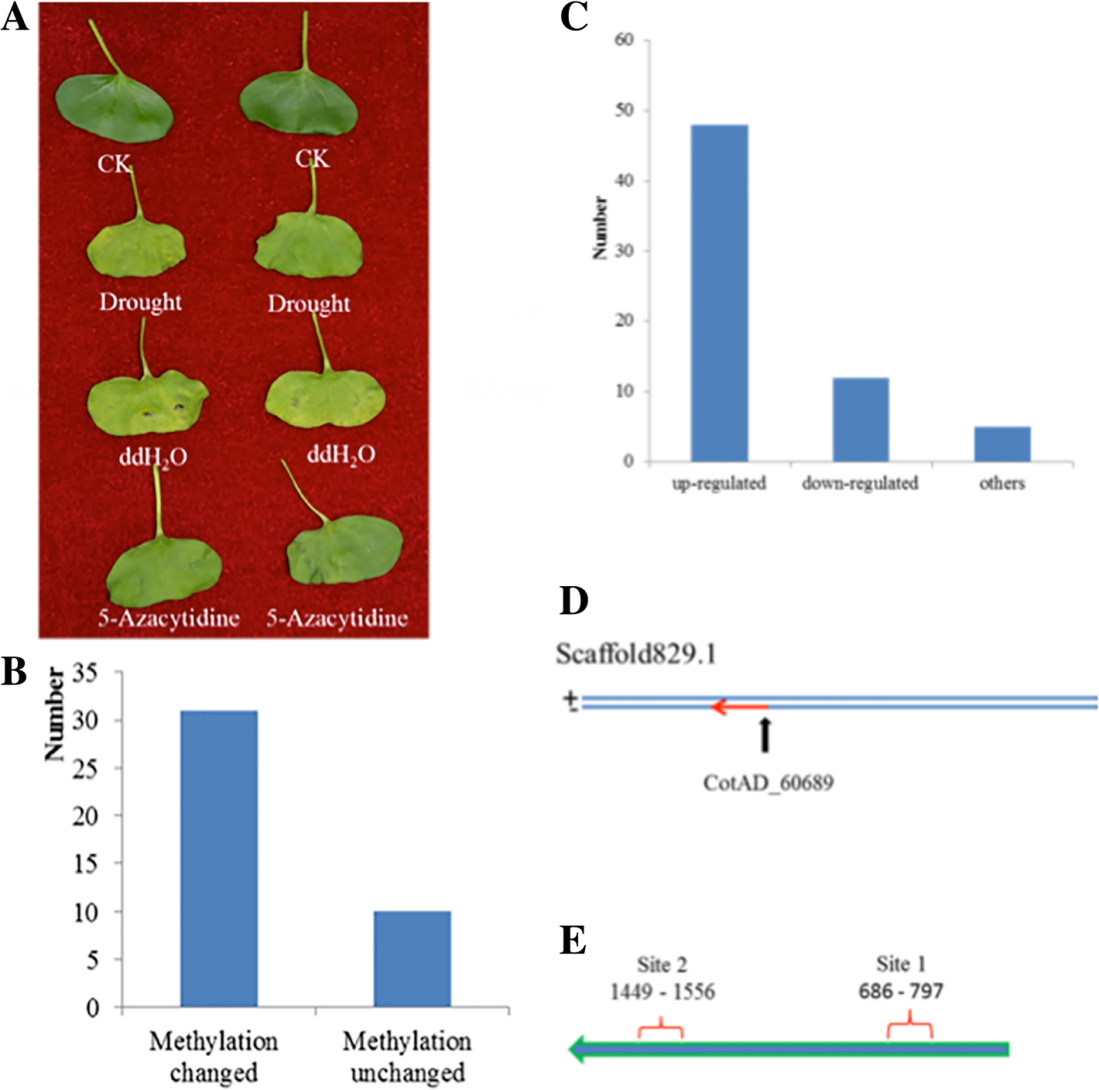
Application of a methylation inhibitor to enhance drought resistance. **a** Morphological changes of cotyledons between CK and 5-Azacytidine-treated under drought stress. CK represents cotyledons without any treatments; Drought represents cotyledons under drought stress; ddH_2_O and 5-Azacytidine represent cotyledons injected with ddH_2_O and 5-Azacytidine, respectively. Two cotyledons from each treatment were selected and all showed a similar symptom. **b** Number of genes methylation state changed of 41 hormone- related genes. **c** Expression analysis of plant hormone-related genes with qRT-PCR. **d** The location of two Auxin-related genes. **e** Prediction of methylation sites of target gene CotAD_60689. Criteria: size > 100, GC Percent > 50.0, Obs/Exp > 0.6

It has been supposed that DNA methylation contributes to the response to drought stress in cotton because the alterations in methylation status would change the transcription and expression of hormone-related genes. To determine whether the methylation changes in these genes participate in regulating the response to drought (and because no methyltransferase mutants are available in cotton), we injected 5-azacytidine, a general inhibitor of DNA (mainly 5-cytosines) methyltransferases, into cotyledons before drought stress treatment and discovered improved resistance in the treated plants. The cotyledons of the 5-azacytidine-treated cotton plants were all still green, while most of the untreated or dd_2_O-treated cotyledons turned yellow and even began to fall off under the stress (Fig. 6a). Gene expression analysis revealed the substantial decrease in the methyltransferase-related lncRNAs XLOC_181940, XLOC_005742 and XLOC_289283 and the sharp increase in their targets, CotAD_50357, CotAD_14650 and CotAD_72292, which also confirmed the reliability of the method. Furthermore, the methylation-specific PCR (MSP) results confirmed that 75.38% (49/65) of the hormone-related genes identified were induced to methylation changes (Fig. 6b), of which 66.7% (4/6), 77.78% (7/9), 50% (8/16) and 52.94% (18/34) of the genes related to ethylene, gibberellin, cytokinin and auxin, respectively, were de-methylated. Expression analysis showed that more genes presented an up-regulated trend by a large margin (Fig. 6c). Interestingly, one gene (named CotAD_60689) associated with auxin was discovered to be regulated through hyper- or hypo-methylation changes, and the position of the gene is shown in Fig. 6d. Next, we conducted methylation site prediction for CotAD_60689, and two sites were found (site 1 was located at 686–697, and site 2 was located at 1449–1556) (Fig. 6e). Based on the observations that  more than two-thirds (46/65) of hormone-related genes epigenetically changed under drought stress and that  methylation inhibitor treatment could enhance the drought tolerance by delaying the speed of falling and turning yellow of cotyledons, we proposed that the inhibition of DNA methyltransferase removes the transcript and expression constraint that prevents massive expression of some hormone-related genes during the drought stress response.

## Discussion

Drought stress, together with other adversities, such as salt, cold and biotic factors, generates serious challenges to plant growth and development. Hence, plants have developed remarkable capabilities to modulate the physiological and molecular machinery through genome-wide gene-expression changes in response to these environmental perturbations [37]. This study provides insights into the potential of a dynamic epigenome in the drought response in *Gossypium hirsutum* L. Our data showed that drought stress could induce an up-regulated epigenome, in which three sequence contexts, including ^m^CpG, ^m^CHG, ^m^CHH contexts, all showed a hyper-methylation pattern after drought stress, which was consistent with previous research [38]. Furthermore, the methylation level decreased to some extent but was still slightly higher in re-watered cotton seedlings than in the control, which suggested that some methylation variations could be retained by memory and even inherited by the next few generations and that many methylation variations changed with the treatments; when the stress was removed, these methylation sites could be restored to their original state. Thus, drought-induced epigenetic changes in the cotton genome can be considered a very important regulatory mechanism for cotton plants to adapt to drought and possibly other environmental stresses. Moreover, more significant changes were found in asymmetric CHH contexts than in CG and CHG contexts, changing dynamically with the external environments, which suggested that CHH methylation may be mostly correlated with environments. Publications have shown that CHH DNA methylation/demethylation may constitute a potential novel epigenetic modification mechanism that regulates growth performance in higher plants over the stress period [39]. Thus, we deduced that methylation/demethylation in CHH contexts may be correlated with external environments and different growth stages, comprising a complex epigenetic regulatory pathway with other epigenetic modifications, such as histone acetylation and histone methylation.

Based on the fact that DNA methylation is always associated with nucleotide sequences [40], we found that the preference for DNA methylation is possibly associated with the symmetric sequence context of CG and CHG and sequence regions with a high or low density of DNA methylation. Many lines of evidence have proven that epigenetic modifications, such as DNA methylation and histone modification, play a crucial role in regulating gene expression in response to various environmental stresses in plants [41, 42]. DNA methylation state alterations in different regions (promoters, exons and introns) cause different gene expression changes. Unlike the CG context and the CHG context, CHH context methylation in promoter regions presents hyper-methylation under drought stress and then recovers to normal levels, which shows that CHH context methylation dynamically changes and is most closely related to the environments. Publications have proven that methylation in the CHH context promotes de novo methylation on flanking intergenic chromatin (particularly within 1 kb of gene starts and ends) [43]. Thus, the disproportionally high levels of CHH relative to CG and CHG that we found in this study suggested that a skewed ratio of de novo methylation near genes would be beneficial for the drought response. Furthermore, methylation alterations in promoter regions would lead to a greater change in expression than those in other gene elements, such as exons and introns, which may be because methylation in promoter regions would influence the transcription of genes with microRNAs and RNA polymerases. Studies in humans have also shown that DNA methylation is an important epigenetic mechanism for gene silencing and cancer progression and that aberrant methylation is mainly found in CpG dinucleotides within promoter regions, which is an important pathway for the repression of gene transcription [44, 45].

**Fig. 7:**
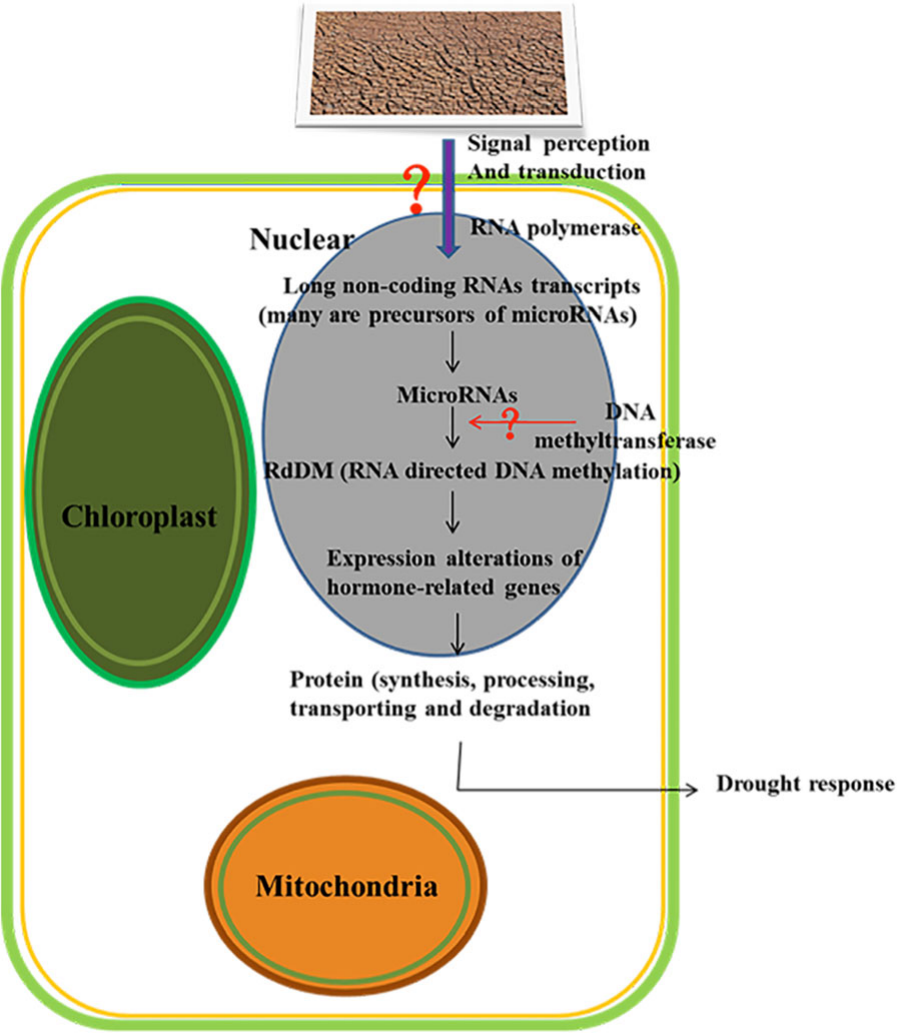
Model of regulation of hormone-related genes in drought response

On the basis of our observations that (1) the methylation status of 41 selected hormone-related genes changed according to the external environment (ck, drought and re-watering), (2) the hormone content and gene expression level of these 41 genes also changed with treatment by drought and re-watering, and (3) methylation inhibitor injection induced the enhancement of drought tolerance and the alterations of methylation status, we proposed that the methylation status of hormone-related genes is one of the mechanisms responding to drought stress. Studies have shown that hormones (proline, ABA, ZR, ETH and AUX, etc.) are associated with drought tolerance and that selecting and using cultivars with a higher proline, ABA, ZR and AUX content under drought stress would be a practical approach to improve drought tolerance in plants [46]. Expression analysis of these 41 hormone-related genes in the investigation also confirmed these results. Combined with the expression data of these hormone-related genes, the long non-coding RNAs and microRNAs, we inferred that long non-coding RNAs would regulate the occurrence of methylation by splicing into microRNAs (RNA-directed DNA methylation, RdDM) in hormone content regulation in response to drought stress. Karakulah et al. have reported that both lncRNAs and microRNAs could block the binding of specific transcript leading to increase mRNA expression by a newly discovered regulatory mechanism called endogenous target mimicry (eTM) [47]. Based on our results and those of previous studies, we propose a four-component model for hormone regulation, in which, through interacting mechanisms that remain unclear, long non-coding RNAs can direct DNA methylation through splicing into microRNAs in response to drought stress (Fig. 7). Angiosperms seem to use their epigenomic stability as a supplemental regulatory mechanism controlling a developmental transition that could cause severe energy and substance waste if it were to transit from normal growth to response to various stresses.

## Conclusions

This study provides insights into the regulatory network of the dynamic epigenome in response to drought. In addition, previous searches for the “cryptic regulatory role” that promotes responding to stresses and is of significant agricultural value were focused on the expression of stress-tolerance genes, stress-related molecular markers and hormone signaling [48–51]. Indeed, plant breeding projects depend on DNA-base molecular markers (such as QTLs and SNPs) and may overlook much epigenetic variation. Hence, our studies highlight the significance of epigenetic modifications, which are beneficial for future crop-improvement strategies and technologies that consider not only genetic variations but also epigenetic modifications. During the process of responding to drought stress, the DMRs associated with drought tolerance, as well as the hormone-related genes and transcription factors described here, provide an initial set of targets for analyzing epigenomic variations across the identified drought-tolerant cotton varieties and for assessing variations that may form the basis of future expanded selection strategies. Moreover, other pathways, for example, metabolic pathways, signal transduction and ripening, may involve methylation variations, which will require additional experimental data for confirmation.

## Methods

### Sample collection and preparation

Drought-tolerant upland cotton (*Gossypium hirsutum* L.) ZhongH177 seeds were preserved in the Cotton adversity research laboratory (our laboratory) in Institute of Cotton Research of Chinese Academy of Agricultural Sciences for many years. The seeds were sterilized with 0.1% HgCl_2_ and placed in a sterile culture dish to accelerate germination before planting into the sand. Cotton seedlings with uniform size were planted into a sand container (10 seedlings per container) in the greenhouse (14 h/day, 30 °C and 10 h/night, 24 °C) of the Institute of Cotton Research of Chinese Academy of Agricultural Sciences. At trefoil stage (three true leaves), drought stress was conducted by withholding watering till the relative water content (RWC) in pots reaches to about 7.0%, while the control pots were watered as before. For methyltransferase inhibitor treatment, 500 μl (a small part of which would be wasted in the injection process) of 1 mM 5-azacytidine aqueous solution was injected into the cotyledons. Then the second and third true leaves from ten plants were harvested together, snap frozen with liquid nitrogen, and stored at −80 °C until use. The method used for sampling was the same for both the control and treatment samples. And the long non-coding RNA sequencing was repeated with three times and whole-genome bisulfite sequencing was conducted with the sequencing depth was 30×.

### DNA, RNA isolation, quantification and qualification

Genomic DNA and RNA were extracted with CTAB method [52]. Genomic DNA and RNA degradation and contamination were monitored on 1% agarose gels. DNA and RNA purity were checked using the NanoPhotometer spectrophotometer (IMPLEN, CA, USA). DNA concentration was measured using Qubit DNA Assay Kit in Qubit 2.0 Flurometer (Life Technologies, CA, USA).

### Library preparation and quantification

A total amount of 5.2 microgram genomic DNA spiked with 26 ng lambda DNA were fragmented by sonication to 200–300 bp with Covaris S220, followed by end repair and adenylation. Cytosine-methylated barcodes were ligated to sonicated DNA as per manufacturer’s instructions. Then these DNA fragments were treated twice with bisulfite using EZ DNA Methylation-Gold Kit (Zymo Research). And the resulting single-strand DNA fragments were PCR amplificated using KAPA HiFi HotStart Uracil + Ready Mix (2X). Library concentration was quantified by Qubit 2.0 Flurometer (Life Technologies, CA, USA) and quantitative PCR, and the insert size was checked on Agilent Bio-analyzer 2100 system. The method of lncRNAs library construction, sequencing, data analysis were shown as Lu et al. [53].

### Clustering and sequencing

The clustering of the index-coded samples was performed on a cBot Cluster Generation System using TruSeq PE Cluster Kit v3-cBot-HS (Illumia) according to the manufacturer’s instructions. After cluster generation, the library preparations were sequenced on an Illumina Hiseq 2000/2500 platform and 100 bp/50 bp single-end reads were generated. Image analysis and base calling were performed with the standard Illumina pipeline, and finally 100 bp paired-end reads were generated.

### Data analysis

#### Quality control

Read sequences produced by the Illumina pipeline in FastQ format were first pre-processed through in-house perl scripts. Firstly, as a subset of reads contained all of part of the 3’adapter oligonucleotide sequence, every read was scanned for the adapter sequence, and if detected the read was filtered out. Secondly, since some reads had N (unknown base) in their sequences, the percentage of Ns in each read was calculated, and if the percentage of Ns was larger than 10% the read was removed. Thirdly, reads with low quality (PHRED score ≤ 5, and percentage of the low quality bases ≥ 50%) were trimmed. At the same time, Q20, Q30andGC content of the data were calculated. The remaining reads that passed the filters were called as clean reads and all of the subsequent analyses were based on them.

#### Reads mapping to the reference genome

Bismark software (version 0.12.5) [54] was used to perform alignments of bisulfite-treated reads to a reference genome with the default parameters. The reference genome was firstly transformed into bisulfite-converted version (C-to-T and G-to-A converted) and then indexed using bowtie2 [55]. Sequence reads were also transformed into fully bisulfite-converted versions (C-to-T and G-to-A converted) before they are aligned to similarly converted versions of the genome in a directional manner. Sequence reads that produce a unique best alignment from the two alignment processes (original top and bottom strand) are then compared to the normal genomic sequence and the methylation state of all cytosine positions in the read is inferred. The same reads that aligned to the same regions of genome were regarded as duplicated ones. The sequencing depth and coverage were summarized using deduplicated reads. The results of methylation extractor were transformed into bigWig format for visualization using IGV browser. The sodium bisulfite non-coversion rate was calculated as the percentage of cytosines sequenced at cytosine reference positions in the lambda genome.

#### Estimating methylation level

To identify the methylation site, we modeled the sum S^+^_i,j_ of methylated counts as a binomial (Bin) random variable with methylation rate r_i, j_

$$ {S^{+}}_{i,\  j} \sim Bin\ \left({S^{+}}_{i,\  j} + {S^{-}}_{i,\  j},\ {r}_{i,\  j}\right) $$

We employed a sliding-window approach, which is conceptually similar to approaches that have been used for bulk BS-Seq (http://www.bioconductor.org/packages/2.13/bioc/html/bsseq.html). With window size w = 3,000 bp and step size 600 bp [56], the sum of methylated and unmethylated read counts in each window were calculated. Methylation level (ML) for each C site shows the fraction of methylated Cs, and is defined as:

$$ ML(C)=\frac{reads(mC)}{reads(mC)+ reads\ (C)} $$

Calculated ML was further corrected with the bisulfite non-conversion rate according to previous studies [57]. Given the bisulfite non-conversion rate r, the corrected ML was estimated as:

$$ ML\ (corrected) = \frac{ML- r}{1- r} $$

#### Differentially methylated regions analysis

Differentially methylated regions (DMRs) were identified using the swDMR software (http://122.228.158.106/swDMR/), which uses a sliding-window approach. The window is setto 1000 bp and step length is 100 bp. Fisher test is implemented to detect the DMRs.

#### GO and KEGG enrichment analysis of DMR-related genes

Gene Ontology (GO) enrichment analysis of genes related to DMRs was implemented by the GO-seq R package [58], in which gene length bias was corrected. GO terms with corrected P-value less than 0.05 were considered significantly enriched by DMR-related genes. KEGG [59] is a database resource for understanding high-level functions and utilities of the biological system, such as the cell, the organism and the ecosystem, from molecular-level information, especially large-scale molecular datasets generated by genome sequencing and other high-through put experimental technologies (http://www.genome.jp/kegg/). We used KOBAS software [60] to test the statistical enrichment of DMR-related genes in KEGG pathways.

#### MicroRNA prediction analysis

Based on the 10,820 long non-coding RNAs, we conducted microRNA prediction analysis with rfam-scan program. Besides, cmsearch program was also used to search homologous microRNAs in database rfam11, and the screening threshold value was E value < =1E-05. And the method used here was the same as the description by Lu et al [49].

#### 5 − Azacytidine treatment

At trefoil stage, 5-azac was diluted with ddH_2_O to 5 mg · ml^−1^. The diluted 5-azaC was injected into cotton true leaves with an injection syringe, as the method described by Zhong. *et al* [11], while the blank control and negative control were performed with nothing and equal amount ddH_2_O, respectively. The injection work was ceased when the liquid spread to 90% area of the leaves. The samples, both the controls and treatment, were harvested when the leaves turned yellow (nearly 24 h).

#### Methylation specific PCR (MSP)

Before carrying out MSP test, DNA bisulfite conversion should be done with DNA bisulfite conversion kit (TIANGEN). In which, Bisulfite mix should be prepared firstly and a 120 μl volume system, containing DNA 3.3 μl (~300 ng•μl-1), Bisulfite mix 90 μl and ddH_2_O 26.7 μl, was used in the conversion process. After the preparation, the program used in the reaction was 95 °C, 10 min, then 64 °C, 60 min, then 4 °C, forever. If DNA content was < 500 ng, time could be shorten to 30 min at 64 °C, treatment would be all right. If DNA content was > 500 ng, 60 min should be used. After the bisulfite conversion, bisulfite-treated DNA should be purified as the direction.

Purified bisulfite-treated DNA was used as templates for methylation specific PCR (MSP), and MSP primers were designed with online program (http://www.urogene.org/cgi-bin/methprimer/methprimer.cgi) [61]. 20 μl volume system, containing template DNA 3.5 μl (~100 ng•μl-1), forward primer 1 μl (10 μM), reverse primer 1 μl (10 μM), dNTPs 1.6 μl (2.5 mM), MSP DNA polymerase 0.5 μl (2.5 U · μl-1), 10 × PCR buffer 2 μl and ddH2O 10.3 μl was used. Program in the MSP reaction was 95 °C, 5 min, 94 °C, 20 s, 60 °C, 30 s, 72 °C, 20 s, 72 °C, 5 min, 35 cycles. After the MSP reaction, 10 μl PCR production was used to detection by 1% agarose gel electrophoresis.

#### Hormone content determination

Plant hormones content determination method was shown as Lu et al [53].

## Additional files

Additional file 1: Figure S1. Distribution of methylation sites in each chromosome in control sample (PNG 1360 kb)

Additional file 2: Figure S2. Distribution of methylation sites in each chromosome in drought-treated sample (PNG 1385 kb)

Additional file 3: Figure S3. Distribution of methylation sites in each chromosome in re-watered sample3 (PNG 1367 kb)

Additional file 4: Figure S4. DMRs length analysis in each chromosome between drought and control sample (PNG 107 kb)

Additional file 5: Figure S5. DMRs length analysis in each chromosome between re-watering and drought sample (PNG 103 kb)

Additional file 6: Figure S6. DMRs length analysis in each chromosome between re-watering and control sample (PNG 108 kb)

Additional file 7: Figure S7. GO analysis of hyper-methylated genes associated with drought (PNG 111 kb)

Additional file 8: Figure S8. GO analysis of hypo-methylated genes associated with drought (PNG 102 kb)

Additional file 9: Figure S9. Statistics of Pathway Enrichment of differentially methylated genes (PDF 6 kb)

## Abbreviations

DMCs: Differentially methylated cytosines
DMRs: Differentially methylated regions
eTM: Endogenous target mimicry
LncRNAs: Long non-coding RNAs
MSP: Methylation-specific PCR
NcRNAs: Non-coding RNAs
RdDM: RNA-directed DNA methylation
RWC: Relative water content
WGBS: Whole-genome bisulfite sequencing
