# Supplementary figures and images for: Single-base resolution methylomes of upland cotton (Gossypium hirsutum L.) reveal epigenome modifications in response to drought stress
Source: BMC Genomics. 2017 Apr 13;18:297. doi: 10.1186/s12864-017-3681-y (PMC5390369; doi:10.1186/s12864-017-3681-y)

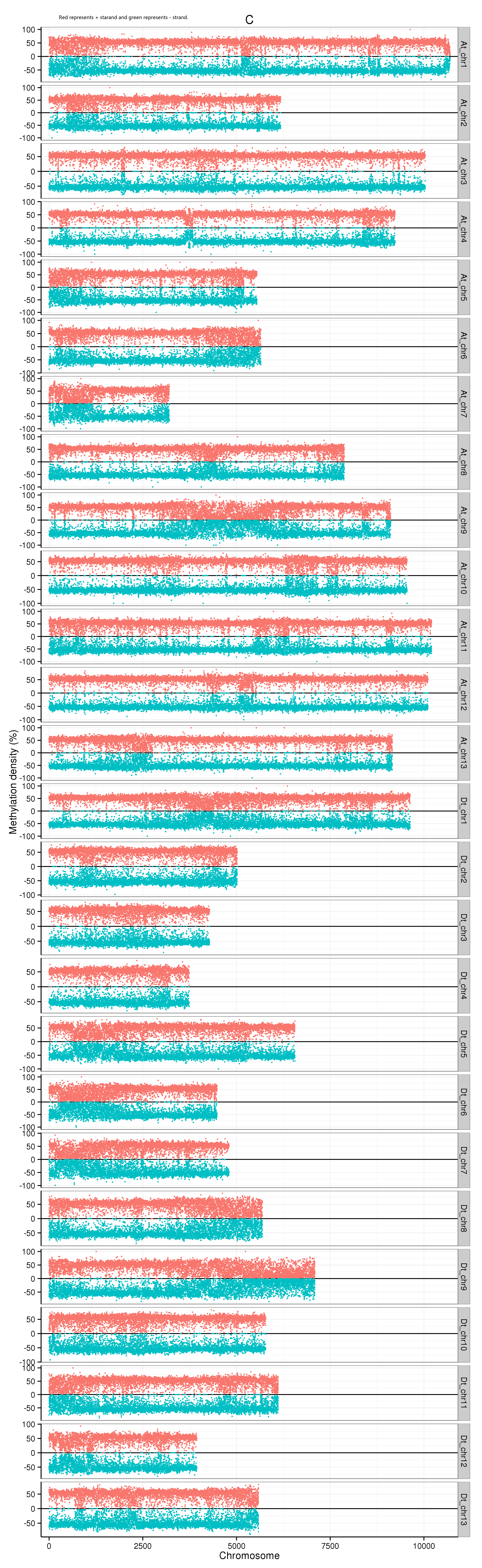

Supplement: Supplementary file 1 — Distribution of methylation sites in each chromosome in control sample (PNG 1360 kb) [file 12864_2017_3681_MOESM1_ESM.png]

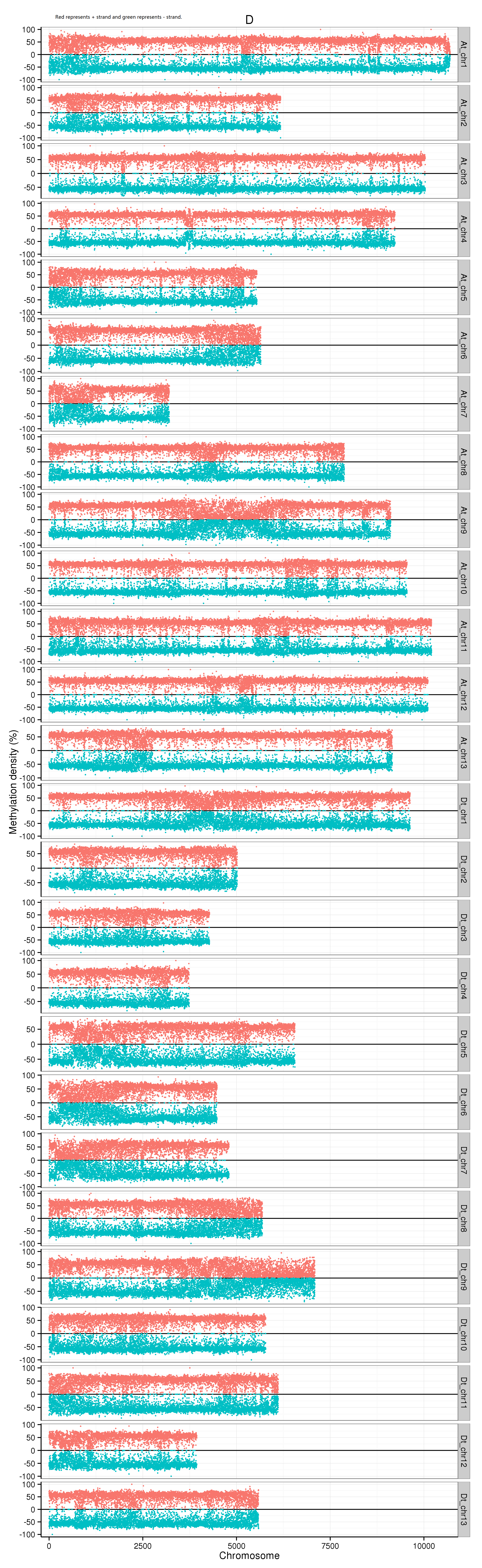

Supplement: Supplementary file 2 — Distribution of methylation sites in each chromosome in drought-treated sample (PNG 1385 kb) [file 12864_2017_3681_MOESM2_ESM.png]

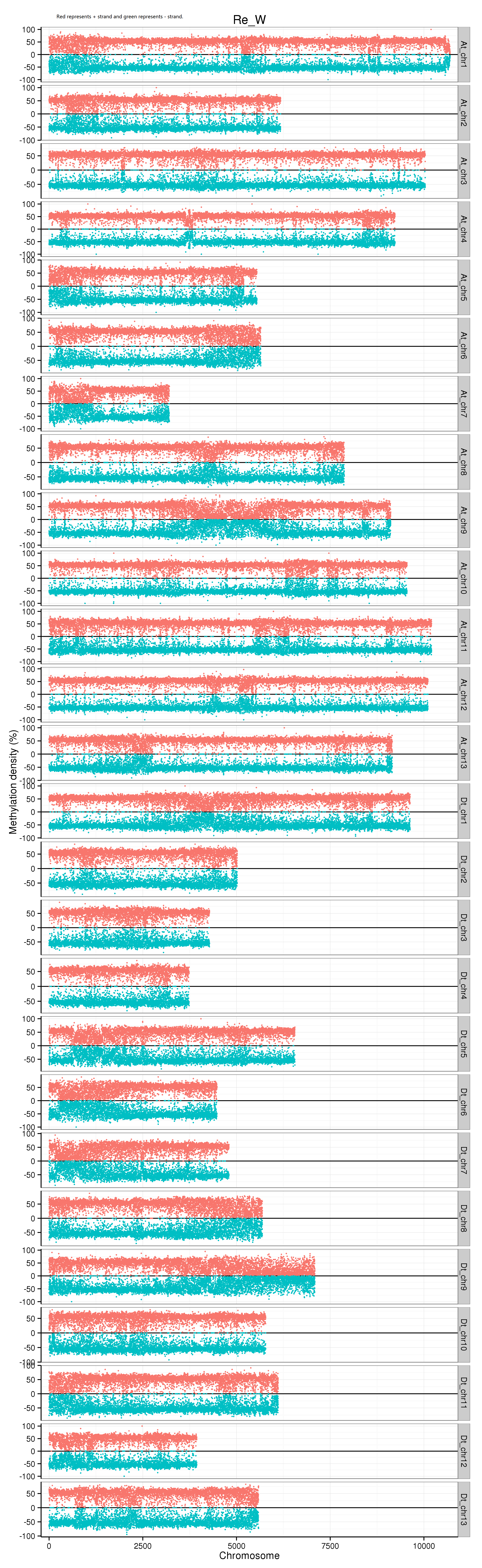

Supplement: Supplementary file 3 — Distribution of methylation sites in each chromosome in re-watered sample3 (PNG 1367 kb) [file 12864_2017_3681_MOESM3_ESM.png]

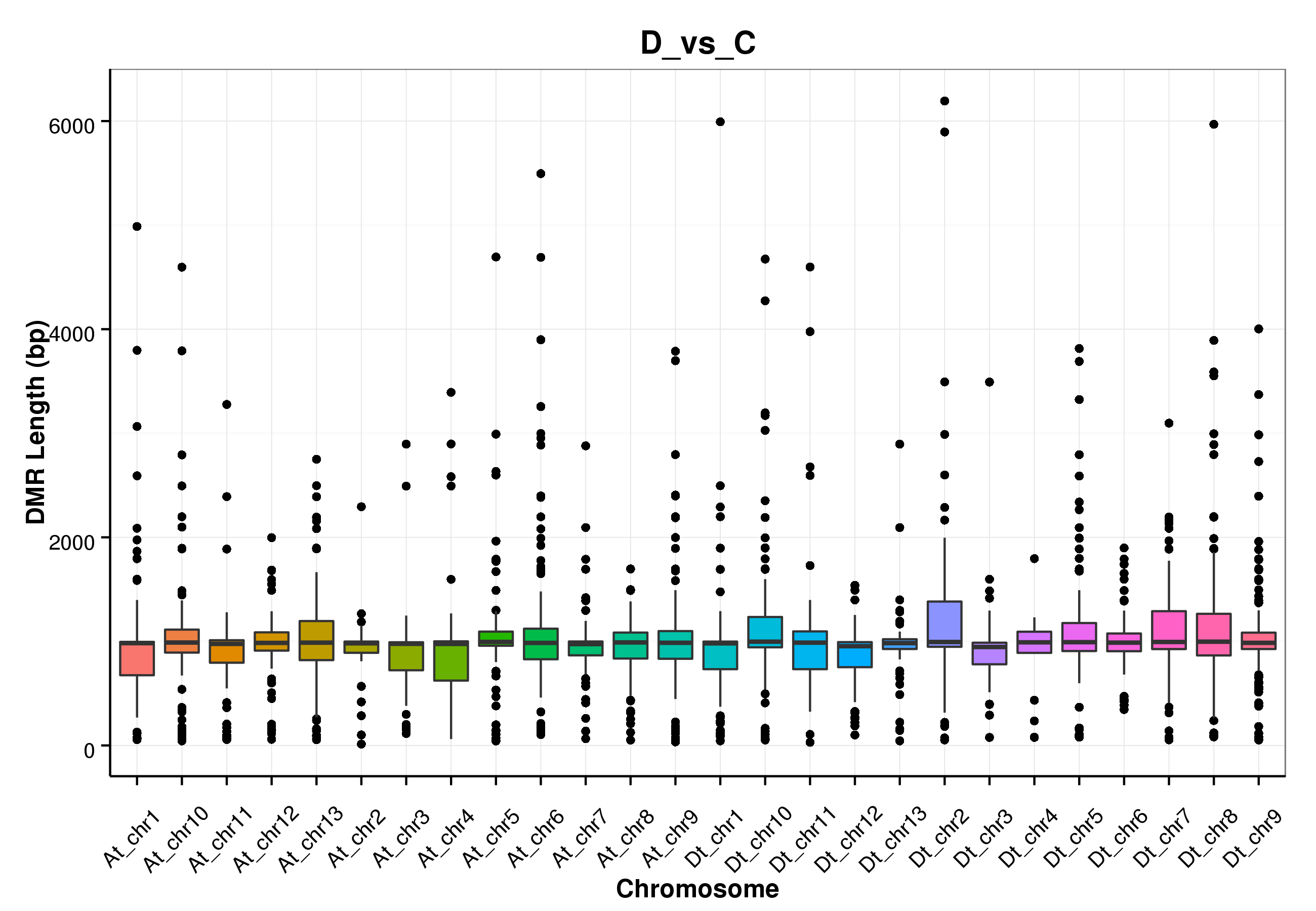

Supplement: Supplementary file 4 — DMRs length analysis in each chromosome between drought and control sample (PNG 107 kb) [file 12864_2017_3681_MOESM4_ESM.png]

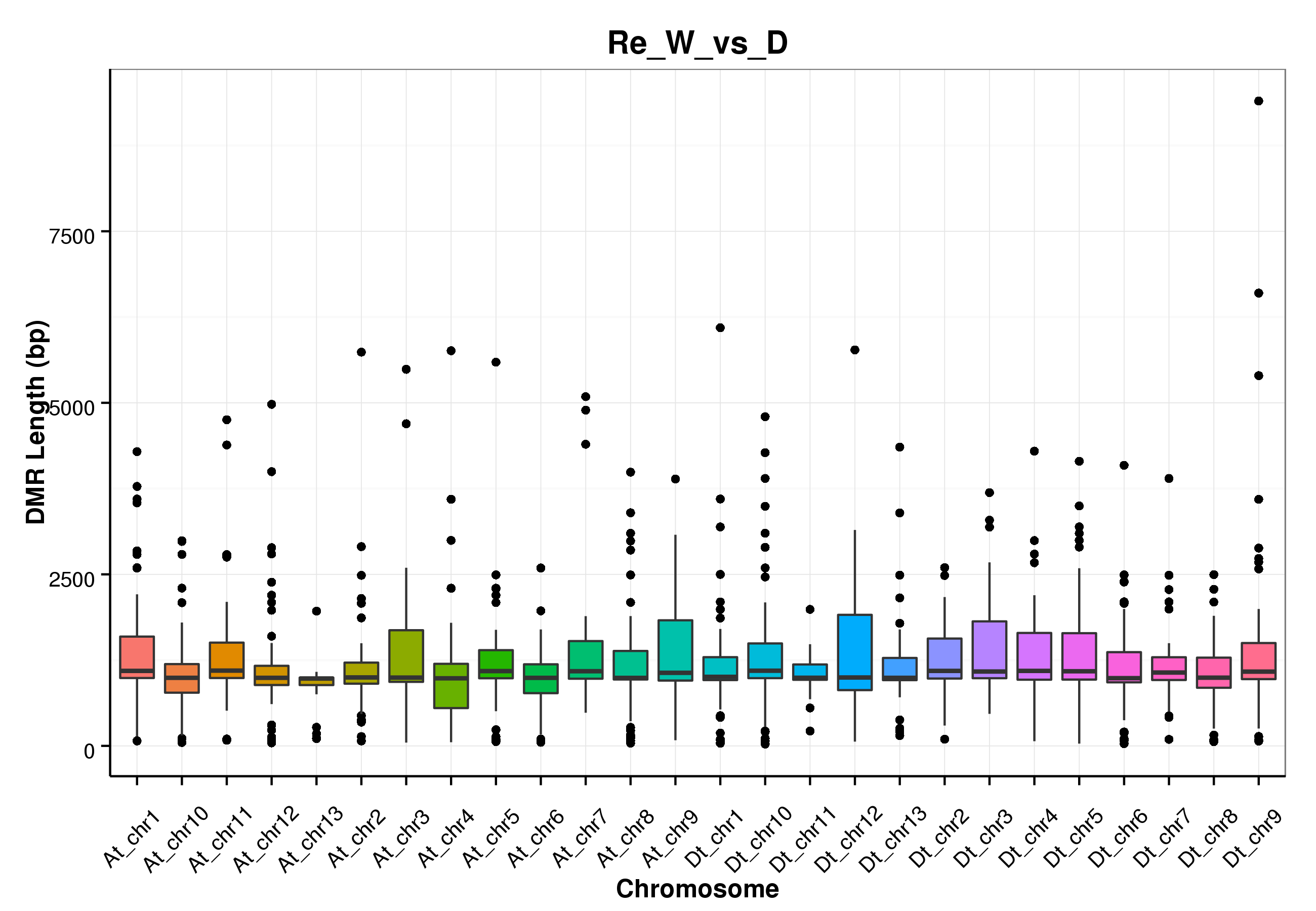

Supplement: Supplementary file 5 — DMRs length analysis in each chromosome between re-watering and drought sample (PNG 103 kb) [file 12864_2017_3681_MOESM5_ESM.png]

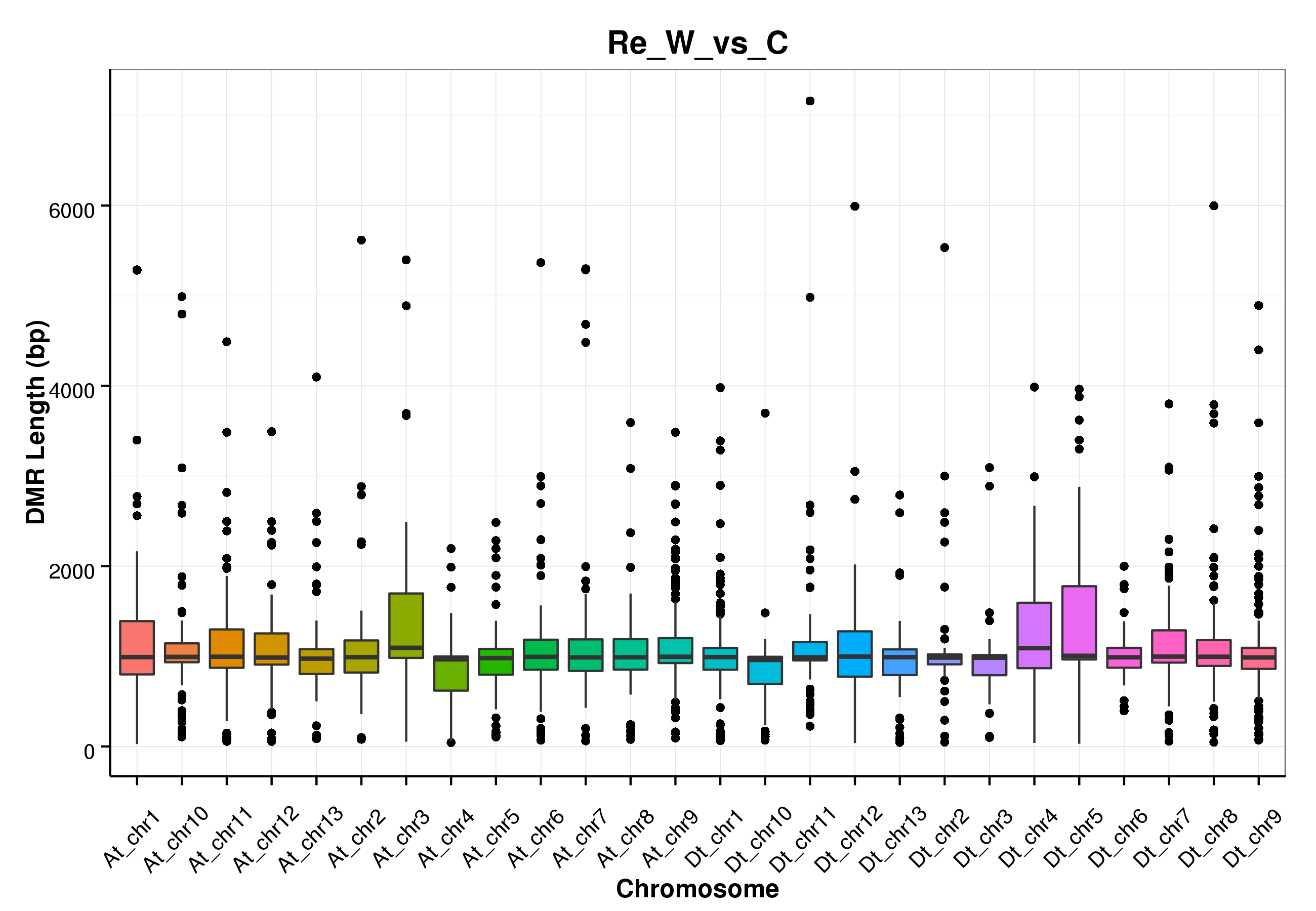

Supplement: Supplementary file 6 — DMRs length analysis in each chromosome between re-watering and control sample (PNG 108 kb) [file 12864_2017_3681_MOESM6_ESM.png]

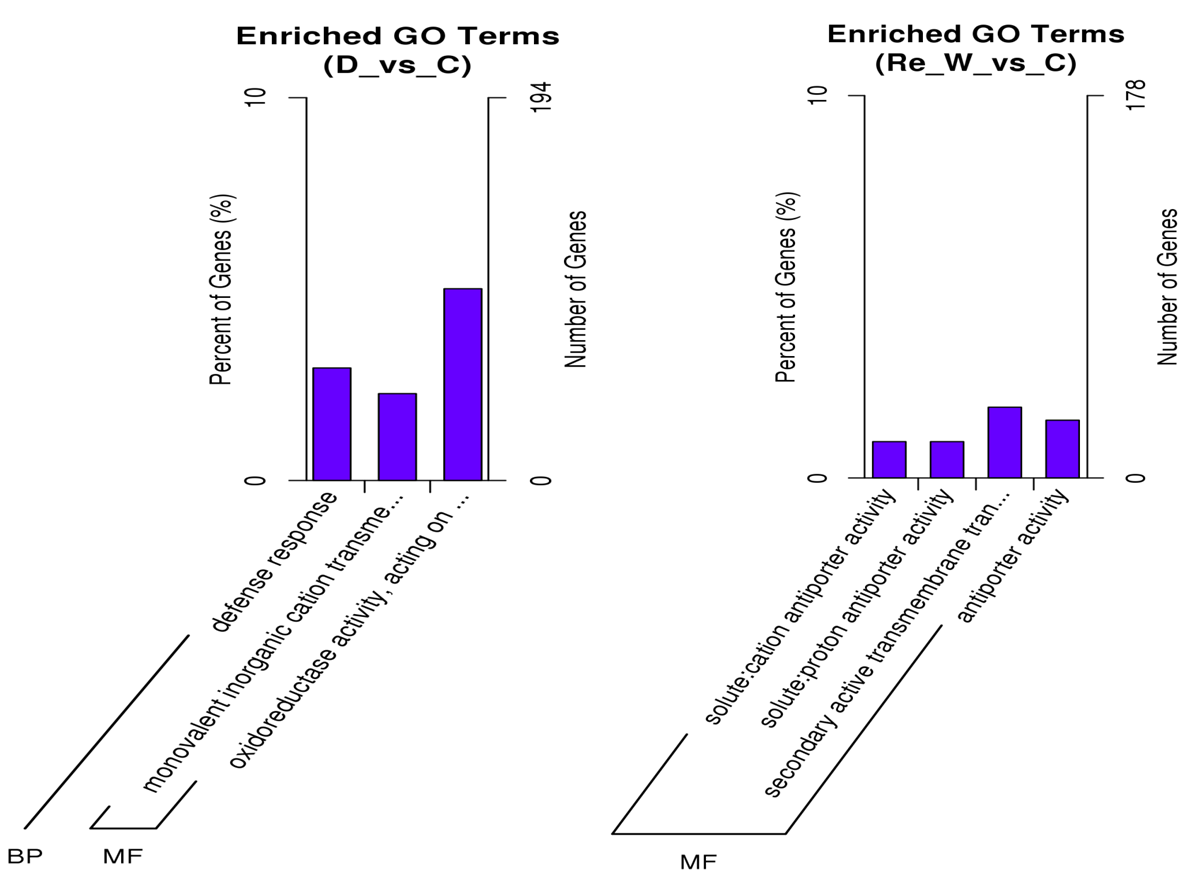

Supplement: Supplementary file 7 — GO analysis of hyper-methylated genes associated with drought (PNG 111 kb) [file 12864_2017_3681_MOESM7_ESM.png]

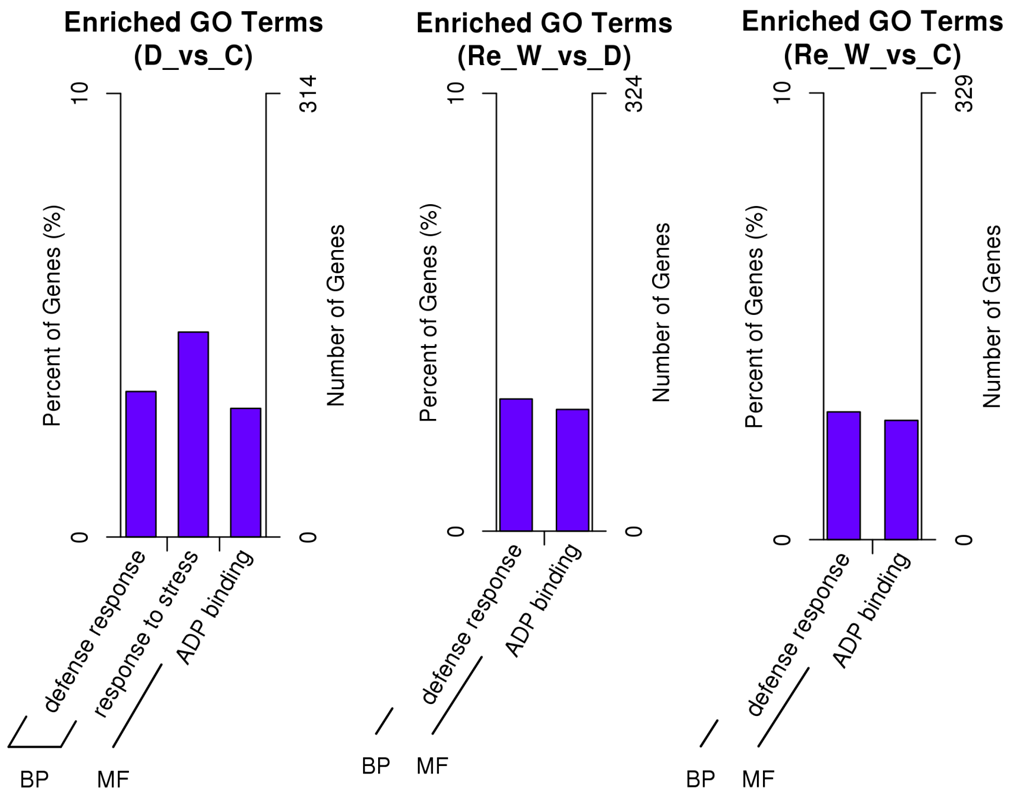

Supplement: Supplementary file 8 — GO analysis of hypo-methylated genes associated with drought (PNG 102 kb) [file 12864_2017_3681_MOESM8_ESM.png]

# Statistics of Pathway Enrichment

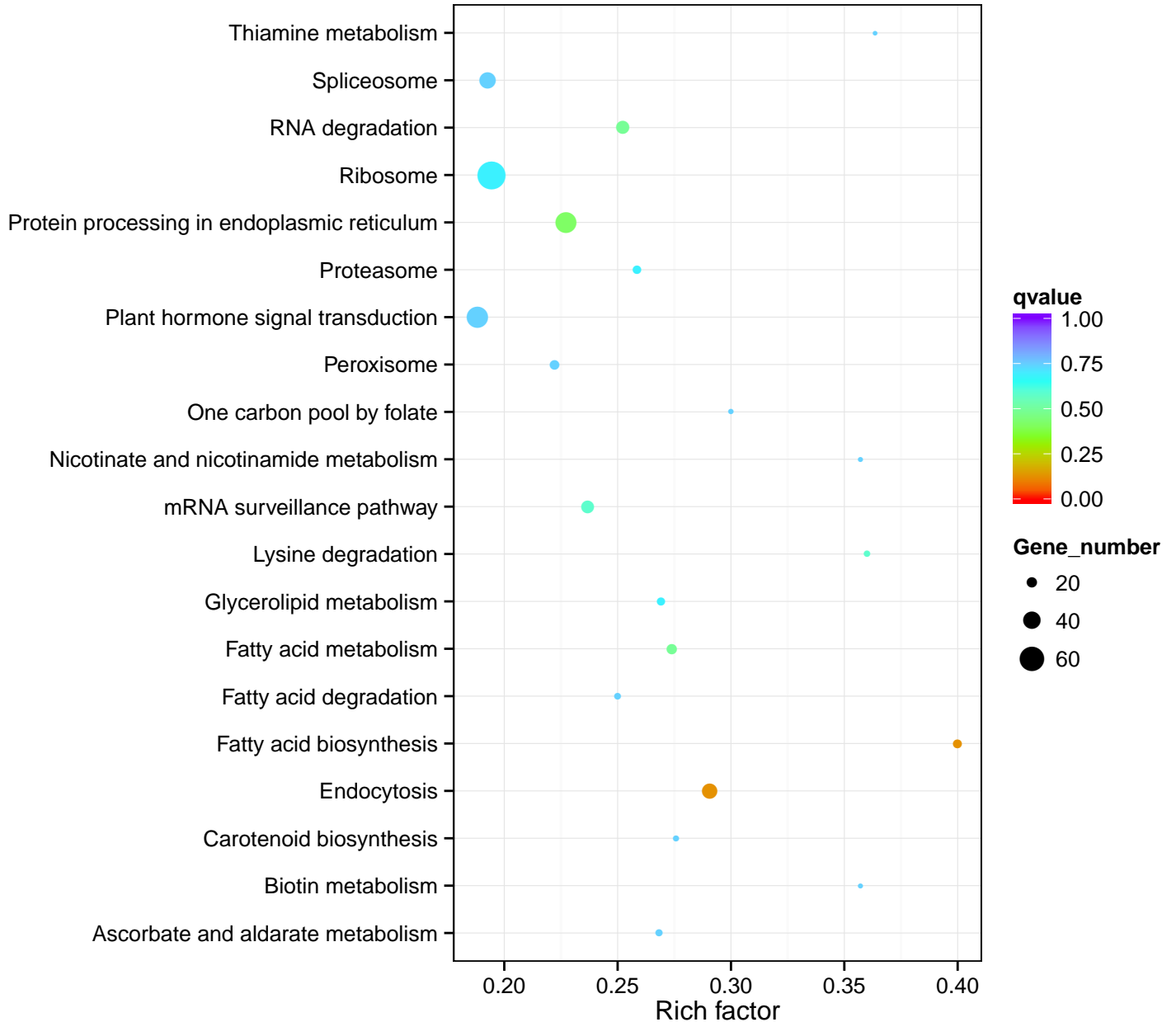

Supplement: Supplementary file 9 — Statistics of Pathway Enrichment of differentially methylated genes (PDF 6 kb) [file 12864_2017_3681_MOESM9_ESM.pdf]
